# Characterization of Eighty-Eight Single-Nucleotide Polymorphism Markers in the Manila Clam *Ruditapes philippinarum* Based on High-Resolution Melting (HRM) Analysis

**DOI:** 10.3390/ani14040542

**Published:** 2024-02-06

**Authors:** Sichen Zheng, Yancui Chen, Biao Wu, Liqing Zhou, Zhihong Liu, Tianshi Zhang, Xiujun Sun

**Affiliations:** 1 State Key Laboratory of Mariculture Biobreeding and Sustainable Goods, Yellow Sea Fisheries Research Institute, Chinese Academy of Fishery Sciences, Qingdao 266071, China; zhengsichen1019@163.com (S.Z.); wubiao@ysfri.ac.cn (B.W.); zhoulq@ysfri.ac.cn (L.Z.); liuzh@ysfri.ac.cn (Z.L.); 2 Laboratory for Marine Fisheries Science and Food Production Processes, Laoshan Laboratory, Qingdao 266237, China; 3 College of Fisheries and Life Science, Shanghai Ocean University, Shanghai 201306, China; 4 Zhangzhou Aquatic Technology Promotion Station, Zhangzhou 363000, China; 15260121656@163.com

**Keywords:** *Ruditapes philippinarum*, RAD-seq, SNP, high-resolution melting

## Abstract

**Simple Summary:**

In this study, we performed a simplified genome sequencing of 301 individuals from 10 populations of *Ruditapes philippinarum* in coastal China using restriction site-associated DNA sequencing (RAD-seq) technology, and obtained a large number of single-nucleotide polymorphism (SNP) markers of Manila clam. Eighty-eight SNP markers were successfully developed using high-resolution melting (HRM) analysis, and the genetic structure and genetic diversity of two populations were analyzed. SNP markers provide a valuable resource for population and conservation genetics studies of this commercially important species.

**Abstract:**

Single-nucleotide polymorphisms (SNPs) are the most commonly used DNA markers in population genetic studies. We used the Illumina HiSeq4000 platform to develop single-nucleotide polymorphism (SNP) markers for Manila clam *Ruditapes philippinarum* using restriction site-associated DNA sequencing (RAD-seq) genotyping. Eighty-eight SNP markers were successfully developed by using high-resolution melting (HRM) analysis, with a success rate of 44%. SNP markers were analyzed for genetic diversity in two clam populations. The observed heterozygosity per locus ranged from 0 to 0.9515, while the expected heterozygosity per locus ranged from 0.0629 to 0.4997. The value of F_IS_ was estimated to be from −0.9643 to 1.0000. The global F_st_ value was 0.1248 (*p* < 0.001). After Bonferroni correction, 15 loci deviated significantly from the Hardy–Weinberg equilibrium (*p* < 0.0006). These SNP markers provide a valuable resource for population and conservation genetics studies in this commercially important species.

## 1. Introduction

Aquaculture plays a key role in global food security. It is predicted that aquaculture will provide vital food and nutrition for more than nine billion people by the middle of the twenty-first century [1]. However, the rapid and sustainable development of aquaculture is hampered by a number of factors, including inbreeding depression, various environmental pressures, and emerging pathogens or diseases [2]. Selective breeding is, therefore, increasingly recognized as a key way of the sustainable production of aquaculture species [3]. Aquaculture breeding programs aim to improve production efficiency to meet consumer demand and increase commercial profits [2]. Characterizing the genetic basis of economically important traits helps to improve the production efficiency of aquaculture species [4]. Aquaculture breeding programs tend to lag behind terrestrial livestock breeding programs in the adoption of genomic technologies [5]. However, the rapid development of next-generation sequencing (NGS) technologies now allows for the rapid and accurate generation of a wide range of genomic resources for any organism at an affordable cost, providing support for the detection of genome-wide highly informative markers in natural and captive populations of both model and non-model species [6]. Thus, NGS technologies provide an opportunity for emerging aquaculture species or species lacking genomic resources by enabling the rapid generation of genome-wide markers for omics-level analysis [7]. Restriction-site associated DNA sequencing (RAD-Seq) utilizes the Illumina next-generation sequencing platform to simultaneously assay hundreds of individuals, resulting in the evaluation and scoring of hundreds of thousands of SNP markers [8]. It has been reported in aquaculture species such as Eastern Oyster *Crassostrea virginica*, Pacific oysters *Crassostrea gigas*, and rainbow trout *Oncorhynchus mykiss* [9,10,11]. SNP markers are playing an increasingly important role in family identification, the genetic analysis of wild and selected populations, the construction of high-density genetic maps, and the fine mapping of quantitative trait loci [12].

High-throughput SNP screening and marker development rely on efficient genotyping platforms [13]. High-resolution melting (HRM) curve analysis has been shown to be a highly sensitive method for mutation discovery and SNP genotyping [12]. The HRM-based SNP genotyping method is based on the measurement of fluorescence changes accompanied by double-strand DNA melting using a saturated DNA intercalating dye. Despite minor variations in DNA sequences, they can also be detected by melting curves, resulting in allele differences among PCR amplicons [14]. At present, the commonly used saturated dyes in HRM analysis are LCGreen, SYTO9, Eva Green, etc., having low inhibition effects on PCR amplification [15]. HRM has many advantages compared with other genotyping methods, such as simplicity, low cost, high sensitivity, and strong specificity, and has been widely used in many non-model species for large- and medium-sized marker development [6].

The Manila clam (*Ruditapes philippinarum*) is an important intertidal marine bivalve, which has become the second most important commercially cultivated bivalve in the world due to its strong resistance to stress, fast growth rates, and fresh meat quality [16,17]. The annual production of Manila clam in China is over 3 million tons, accounting for more than 90% of the world production [18,19]. The genetic diversity and differentiation of *R. philippinarum* will provide the guiding information for the conservation and genetic breeding of clams in China. In recent years, due to the rapid development of large-scale farming and the increase of cultivation areas, the transplant of clams bred in the south to northern areas for culture may have some negative effects on the local population [16,17]. The transplant of clams bred in the south to northern areas for culture may have some negative effects on the genetic structure of the Manila clam, such as the loss of genetic variation, inbreeding depression, and reduced effective population sizes [14]. Therefore, cost-effective SNP genotyping is essential for the natural conservation and genetic breeding of *R. philippinarum*.

In this study, we developed single-nucleotide polymorphism (SNP) markers in *R. philippinarum* by restriction site-associated DNA sequencing (RAD-seq) on the Illumina HiSeq4000 platform. Eighty-eight SNP markers were successfully validated by using high-resolution melting (HRM) analysis. The developed SNP markers were further used for genetic analysis in two clam populations. These findings will provide valuable information for studies on population genetics and the conservation of this commercially important species.

## 2. Materials and Methods

### 2.1. Tissue Sampling

A total of 301 clams (*R. philippinarum*) were collected from the northern and southern coast of China (Table S1). The sampling locations and the number of samples were described in our previous study [14]. The appropriate muscle tissue sample (1 cm^3^) was excised from each clam and immediately stored in 100% molecular grade ethanol at −20 °C until DNA extraction.

### 2.2. DNA Extraction and RAD-seq Library Preparation

The genomic DNA of the samples was extracted using the CTAB (Cetyltrimethylammonium bromide) method. The DNA quality was assessed using Qubit (Thermo Fisher Scientific, Waltham, MA, USA) and Nanodrop (Thermo Fisher Scientific, Waltham, MA, USA) instruments. RAD-seq libraries were constructed following a protocol modified from Baird et al. [20]. Briefly, Genomic DNA (0.1–1 µg; from either individual or pooled samples) was digested with EcoRI (NEB, Ipswich, MA, USA), followed by heat inactivation of the enzyme. The digested fragment was end-repaired and ligated with barcoded P1 adapters using T4 ligase (NEB, Ipswich, MA, USA). Samples were pooled in equal amounts for shearing to an average size of 500 bp. Libraries were size-selected into 300–700 bp fragments by running out on a 1% agarose gel. Libraries were blunt-end-repaired, and a 3′-adenine overhang was added to each fragment. We added a P2 adapter containing unique Illumina barcodes for each library using the NEBNext adapter (NEB, Ipswich, MA, USA). Libraries were amplified through 16 PCR cycles with Phusion high-fidelity DNA polymerase (NEB, Ipswich, MA, USA) and column-purified. Samples were sequenced on an Illumina novaseq6000 (Illumina, San Diego, CA, USA) using 150-bp paired-end reads.

### 2.3. Bioinformatics Analysis

#### 2.3.1. Clean Reads Filtering

Quality trimming is an essential step to generate a high confidence of variant calling. Raw reads were processed to get high-quality clean reads using FASTP [21] according to three stringent filtering standards: (1) removing reads with ≥10% unidentified nucleotides (N); (2) removing reads with >50% bases having phred quality scores of ≤20; (3) removing reads aligned to the barcode adapter.

#### 2.3.2. SNP Identification and Annotation

To identify SNPs, the Burrows–Wheeler aligner (BWA) was used to align the clean reads from each sample against the reference genome with the settings “mem 4-k 32-M”. (-k is the minimum seed length and -M is an option used to mark shorter split alignment hits as secondary alignments) [22]. This genome project has been registered in the NCBI database under the BioProject accession PRJNA929581. The sequencing data have been deposited in the NCBI Sequence Read Archive (SRA) under the accession numbers of SRR23279236, SRR23279237, and SRR23279238 for the genomic data. Repeat sequences generated by PCR amplification during library construction were filtered using Picard software (Picard: http://sourceforge.net/projects/picard/ accessed on 12 December 2022). The Unified Genotyper module in GATK (version 3.4–46) was used for SNP detection (parameter -t Unified Genotyper-glm BOTH -nt 8) -filter “QD < 4.0” || “FS > 50.0” || “MQ < 40.0”, -G_filter “GQ < 20 -filter” Multiallelic (Non allelic genotype) (Text S1) [23]. The detected variants were annotated using ANNOVAR (version 2) [24]. Primer 3 was used to design primers targeting candidate SNPs. The criteria for primer design included a predicted annealing temperature (Tm) of 59 °C to 61 °C, a primer length ranging between 20–24 bp, and PCR amplicon lengths of 100 to 220 bp.

The DNA of 301 *Ruditapes philippinarum* clam individuals was randomly selected as the template and the candidate SNP sites were amplified by PCR reaction to detect the specificity and amplification efficiency of the primers. All PCR reactions were performed in 96-well plates using a 9700 Thermal Cycler (Applied Biosystems, Foster City, CA, USA) in a total volume of 10 µL per well. The PCR reaction mixture consisted of 50 ng of genomic DNA, 0.4 µL of forward and reverse primers, and 2× Taq plus MasterMix II 8 µL (Nanjing Vazyme Biotechnology Co., Ltd., Nanjing, China). After pre-denaturation at 95 °C for 5 min, 40 PCR cycles were performed, including deformation at 94 °C for 30 s, annealing at 56 °C for 10 s, extension at 72 °C for 10 s, and final extension at 72 °C for 5 min. The quality of PCR products was detected by 1.5% agarose gel electrophoresis. Sites with single, bright bands at the corresponding lengths were retained for subsequent analysis.

The SNPs loci obtained from the above screening were now used as templates for HRM on a LightCycler480 fluorescent quantitative PCR instrument (Roche Diagnostics, Burgess Hill, UK) using DNA from three individual clams. Loci with cycle threshold values less than 30 and whose amplification curves had reached a plateau at the end of PCR were retained, and compliant loci were finally used as templates for SNPs polymorphism detection and typing with 32 clam DNAs. The HRM reaction system is as follows. The high-resolution melting curve was performed at the end of ordinary PCR amplification, and the specific procedure was as follows: the PCR products were denatured at 95 °C for 1 min, and then annealed at 40 °C for 1 min to randomly form DNA double helices. Melting curves were generated during warming from 60 °C to 90 °C, and fluorescence intensity data were collected 25 times per 1 °C increase. The software was supplied with LightCycler^®^480 Software 1.5 (Roche Diagnostics) in the gene scanning, as well as the Tm Calling analysis modules for typing SNPs.

### 2.4. Standard Population Genetic Statistics

GenAlEx 6.3 was used to calculate the observed heterozygosity (H_o_), the expected heterozygosity (H_e_), inbreeding coefficient (F_is_), and genetic differentiation coefficient (F_st_). The estimation for the significance of deviations from the Hardy–Weinberg equilibrium (HWE) was also estimated by GenAlEx 6.3 [25]. The minor allele frequency (MAF) was calculated using Genetix 4.05 [26].

## 3. Results

### 3.1. RAD Data Analysis

RAD-tag sequencing generated 658G of raw data for 301 reference individuals before any quality filtering. The number of reads per individual ranged from 640,000 to 37,920,000. After quality filtering, 637G of clean data (with an efficiency of 96.68%) and 4,430,500,632 clean reads were retained. On average, each individual had 14,719,271 (98.49%) sequences retained and 226,557 (1.82%) sequences discarded. Overall, the RAD-seq results demonstrated high Phred quality (Q20 ≥ 95.86%, Q30 ≥ 88.92%) and stable GC content (ranging from 32.51% to 34.49%). Among the retained sequences, on average, 12,114,297 (82.30%) aligned with the Philippine clam genome, with an average of 683,115 (4.64%) single-end aligned sequences and 5,715,591 (38.83%) paired-end aligned sequences. A total of 41,796,316 SNPS were obtained, and 36,661,668 high-quality candidate SNP markers were obtained after GATK filtering.

**Figure 1:**
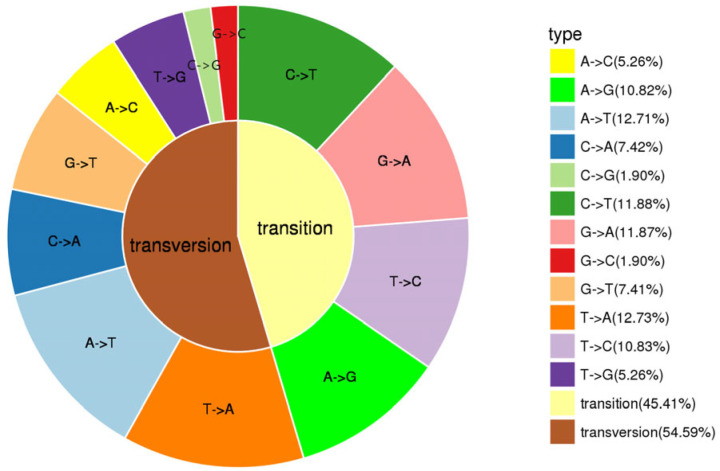
Transitions and transversions occurring in Manila clam *R. philippinarum*.

The point mutations in the SNP typing results can be divided into six types. As shown in Figure 1, A/T, C/T, and A/G were the major mutation types, accounting for 25.44%, 22.71%, and 22.69% of all base mutation types, respectively. A/C and G/T accounted for 12.68% and 12.67%, respectively, while G/C accounted for at least 3.8% of all base mutation types. SNPs were evenly distributed across nucleotide positions of the sequence reads, with no significant increase in SNPs at the end of the reads. In our dataset, about 55% of SNPs were shown to be transversion factors, and the observed ratio of transition factors to transversion factors was 1:1.2 (Figure 1).

### 3.2. Evaluation of SNP Markers for Population Genomic Analysis

**Figure 2:**
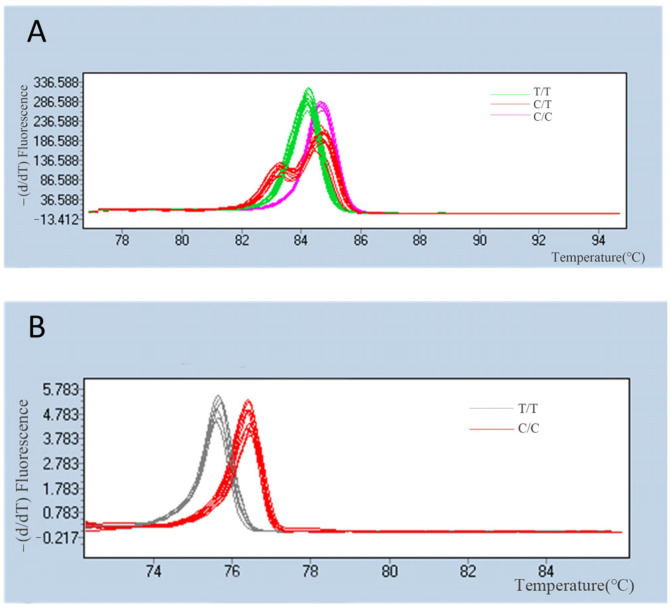
HRM profiles of snp4 and snp5 sites in DY population. (**A**) The green curves represent the TT homozygotes, the purple curves represent the CC homozygotes, and the red curves represent the AG heterozygotes. (**B**) The gray curves represent the TT homozygotes and the red curves represent the CC homozygotes.

In this study, candidate SNP sites were randomly selected, and 200 pairs of primers were designed for PCR amplification. There were 163 pairs (81.5%) of primers with single bright bands and 37 pairs (18.5%) of primers without target bands obtained by agarose gel electrophoresis after PCR amplification. After HRM polymorphism analysis, 88 SNP loci showed polymorphism in the two populations of *Ruditapes philippinarum* clams and were verified, while other loci showed no polymorphism or had chaotic melting curves, which could not be accurately categorized. The success rate of development from 200 pairs of high-quality candidates SNPS was approximately 44%. For instance, the HRM melting curves of two SNP loci (snp4 and snp5) are illustrated in Figure 2, showing the distinct HRM profiles of different genotypes, including homozygotes and heterozygotes.

Of the 200 primer pairs, 88 SNP showed polymorphism in the two populations. The summary statistics of variability of 88 SNP markers in clams are shown in Table S2. The minor allele frequency (MAF) was detected to be from 0.0323 to 0.5000. The observed heterozygosity ranged from 0 to 0.9515, while the expected heterozygosity ranged from 0.0629 to 0.4997. The mean values of observed and expected heterozygosity were 0.3218 and 0.3546, respectively. The value of F_IS_ was estimated to be −0.9643 to 1.0000. Furthermore, 15 of the 88 loci showed significant deviation from the Hardy–Weinberg equilibrium after Bonferroni correction (*p* < 0.0006). The global F_st_ value was estimated to be 0.1248 (*p* < 0.001). The average observed heterozygosity and expected heterozygosity of the two populations were 0.322 and 0.355, respectively. The F_st_ value between DY and WF populations was 0.108. According to AMOVA analysis, the greatest number of variances occurred among individuals (50%), compared to 40% within individuals and 10% among the populations.

## 4. Discussion

A total of 200 SNP loci were randomly selected for validation in this study, and finally, 88 SNP markers were successfully developed in this study, compared to 14 SNP markers developed in the previous study [27]. The success rate of SNP development in this study was about 44%, which is similar to the previous studies on bivalve mollusks such as *C. gigas* (37%) [28] and *Meretrix meretrix* (35%) [29]. As a highly heterozygous species, the presence of SNP loci in the prim-binding region or the poor quality of the flanking sequences of the target SNP loci may lead to the failure of PCR amplification [30,31]. A locus that fails to be genotyped may be due to the amplified product containing more than one SNP. If the amplification region contains two or more than two SNP sites, it will be difficult to identify the accurate genotypes of the melting curves [28]. When two or more SNP sites are present in the amplified product, the dissolution curve generated in HRM analysis will be confused [28]. It is crucial to select primers that are as close as possible to the target SNP for acquiring amplified fragments containing only one SNP. The shorter amplified fragments are more likely to reveal the effects of small sequence variations, which facilitates the differentiation of genotypes using HRM analysis [30]. In addition, the presence of selection pressure and small sample sizes may also affect the efficiency of SNP validation [28,31].

Genetic diversity is an indispensable part of biodiversity, which is composed of the genetic variation of individuals within a species and the variation of different individuals within a population [32]. The mean expected heterozygosity of the DY population was 0.358 and the mean expected heterozygosity of the WF population was 0.352. In the present study, 88 SNP markers were applicable and showed higher polymorphisms in the two clam populations. Similar results have been reported in other mollusks such as the ark-shell *Scapharca subcrenata* (H_e_ = 0.344) and Pacific oyster *C. gigas* (H_e_ = 0.279) [28,33]. According to the present data, the population diversity index of SNP markers, such as expected heterozygosity (H_e_) and observed heterozygosity (H_o_), is lower than that of other molecular markers such as microsatellites [17,34]. This may be caused by the biallelic nature of the existence form of SNP markers. For microsatellite genotyping, some microsatellite loci may have a large number of different genotypes (more than four alleles), while each SNP locus has a maximum of only three genotypes. As such, a larger number needs to be used in population genetics studies of the SNP locus. However, for SNP markers, due to the small number of alleles, the scoring type is fast and the error rate is lower.

As a new generation of molecular marker technology, SNPs are the most common and informative genetic changes in plant and animal genomes, and any base mutations (insertions and deletions) and mutations at sites are not random. Theoretically, there are two mutation types for transition (A↔G or C↔T) and four mutation types for transversion (A↔C, A↔T, G↔C, or G↔T), and the mutation frequency ratio (ts/tv) of transition and transversion should be 1:2, whereas the value of ts/tv in the present study was 1.2, which is similar with that of the previous reports of Pacific oyster (ts/tv = 1.1), American oyster (ts/tv = 1.4), and Atlantic salmon (ts/tv = 1.1). The results showed that C/T was the main base conversion in *R. philippinarum*, which may be due to the methylation of cytosine residues on CpG dinucleotides with a high mutation rate in the genome, and the formation of thymidine through spontaneous deamination, resulting in the relatively higher C/T base conversion. The results were consistent with those of *Chlamys farreri* [35], *C. gigas* [36], and *Mytilus galloprovincialis* [37]. Calculating transition/transversion bias is important for deciphering genome selection and evolution; mutation bias is a driving force of evolution shaped by natural selection in animal kingdom [38]. As reported in the *C. gigas* genome, the proportion of GC -> AT mutations increases with the gene expression level and gene length, while it decreases with the purifying selection acting on amino-acid composition [39].

Genome sequencing is a key step in deciphering molecular mechanisms and accelerating the genetic improvement of important traits in economic species [19]. Although many species’ breeding programs have benefited from new molecular genotyping methods, these advances have been slower in most aquaculture species, especially in bivalves, [40]. With regard to *R. philippinarum*, although the development and use of different types of molecular markers have been carried out over the past two decades, few molecular markers are successfully developed and used in genetic studies, usually less than 20 microsatellite or SNP loci [34,41,42,43]. The present study developed 88 SNP markers by HRM analysis, and further successfully used them for genetic analysis in clam populations. The findings will provide valuable information for providing guiding information on the natural resource conservation and genetic breeding of this commercially important species.

## 5. Conclusions

In this study, more than 36 million putative SNPs were identified according to RAD sequencing data in *R. philippinarum*. Eighty-eight SNP markers were developed and validated by using HRM analysis. The success rate of SNP development in this study was about 44%, which is similar to the previous studies in bivalve mollusks. The developed markers have been used for genetic studies on the two clam populations, indicating their high genetic diversity. In the present study, the development of SNPs not only serves as useful markers for population genetic studies and genetic map construction in *R. philippinarum*, but also provides guiding information on natural resource conservation and genetic breeding for this commercially important species.

## Data Availability

The sequencing data have been deposited in the NCBI Sequence Read Archive (SRA) under the accession numbers of SRR23279236, SRR23279237, and SRR23279238 for the genomic data. The data on sample collection and marker information has been provided in the Supplementary Materials.

## Supplementary Materials

The following supporting information can be downloaded at: https://www.mdpi.com/article/10.3390/ani14040542/s1. Table S1: Sample name, location, and collection date for all populations of *R. philippinarum*. Table S2: Summary statistics of variability at 88 SNP markers in *R*. *philippinarum*. Text S1: GATK Filter description.
