# Supplementary material for: Characterization of Eighty-Eight Single-Nucleotide Polymorphism Markers in the Manila Clam Ruditapes philippinarum Based on High-Resolution Melting (HRM) Analysis
Source: Animals (Basel). 2024 Feb 6;14(4):542. doi: 10.3390/ani14040542 (PMC10886362; doi:10.3390/ani14040542)
Supplement: Supplementary file 1 [file animals-14-00542-s001.zip › animals-2768900-supplementary.pdf]

Table S1. Sample name, location, and collection date for all populations of *R. philippinarum.*

Name	Location	Collection Date
Lianjiang	Lianjiang, Fujian Province	July 2020
Ningbo	Ningbo, Zhejiang Province	July 2020
Sanya	Sanya, Hainan Province	June 2020
Rizhao	Rizhao, Shandong Province	August 2020
Zhangzhou	Zhangzhou, Fujian Province	July 2020
Beihai	Beihai, Guangxi Province	June 2020
Qingdao	Qingdao, Shandong Province	August 2020
Haiyang	Haiyang, Shandong Province	August 2020
Donggang	Donggang, Liaoning Province	August 2020
Zhuanghe	Zhuanghe, Liaoning Province	August 2020

Table S2. Summary statistics of variability at 88 SNP markers in the *Ruditapes philippinarum*.

	Primers	Types	Size	Tm	H_O	H_E	MAF	F_{is}	F_{st}	PHWE
1	F: ACACGTTGCACGATATCCAA<br>R:TTTCAGGAAGCAAGTTTCAATG	A/G	104	59.7	0.0956	0.0909	0.0481	-0.0516	0.0013	1.0000
2	F:AAGCAAATAATGGCGCAGTC<br>R:CCTGCCTCTCTCAACTCCAC	C/T	105	60.1	0.4372	0.4798	0.4000	0.0889	0.0007	0.1368
3	F:TCTGTGTGGACTGTGGCTGT<br>R:GACTGTCGTTGCTGTTCTGG	C/T	106	59.9	0.1563	0.3410	0.2179	0.5418	0.0000	0.0000*
4	F:GGCTCTAGGTTCGACTGACG<br>R:CCTGCTTGTGATGGCACTAA	C/T	109	59.9	0.2286	0.2932	0.3208	0.2203	0.3326	0.1756
5	F:CCCTGGTCTCCACACAGATT<br>R:GTGTTGAGGAAAGCACAGCA	A/G	110	60.0	0.2215	0.4133	0.3333	0.4642	0.0437	0.0012
6	F:ATACCCCAGTGTTGACCAGC<br>R:GTCGGCGTATTGTTACGGTT	A/G	113	59.9	0.1276	0.1499	0.5000	0.1485	0.7001	0.2643
7	F:AATGAGGATGATCAGGGCAG<br>R:AGGCTTACCAACCAACAACG	C/A	114	60.0	0.3740	0.2938	0.1909	-0.2730	0.0337	1.0000
8	F:AAAAACAAATTCACGCCGTC<br>R:GTGCAGTCTCGCCATCTACA	A/G	114	60.0	0.4914	0.4752	0.3909	-0.0340	0.0017	0.6214
9	F:GAGTCTCGAAATGCAAAGCC<br>R:CAAATGCACAACTGGAAACG	C/T	115	60.1	0.0656	0.0629	0.0323	-0.0436	0.0093	1.0000
10	F:CATGTCGTTGTTGGAGCAAG<br>R:GCAACAAGAAACAGTCGCAA	C/A	116	60.2	0.1346	0.2319	0.1442	0.4195	0.0607	0.0052
11	F:AAATAATTGTGTCGGCCAGC<br>R:CCGCAACAGAGAGAATGTCA	G/T	119	60.0	0.1100	0.1039	0.0545	-0.0587	0.0005	1.0000
12	F:TCGAAACTTCCCGATTGTTC<br>R:ACTACAAGAGCCGCCGAGTA	C/A	120	60.0	0.3026	0.2374	0.3919	-0.2750	0.5060	1.0000
13	F:GCACAGTCGCATGAACACTT	C/T	121	60.0	0.6239	0.4862	0.4245	-0.2833	0.0054	0.9636

	R:CCGCTTATGGTTGCAAATCT									
14	F:ACGGCAAGCAAGAAGACAAT<br>R:CCGCCTTACCTGGTGAATTA	G/T	121	59.9	0.2582	0.2245	0.1296	-0.1502	0.0017	1.0000
15	F:CCTCTCTGCCATTGTTTTCC<br>R:AAAGCAAAAGAAAAAGGCCA	C/T	127	59.3	0.0750	0.4688	0.3750	0.8400	0.0000	0.0000*
16	F:CCTGGTCAAGTCTCGTCACA<br>R:TGCTCTTTCTCGGACACTGA	C/T	127	59.8	0.4375	0.4579	0.3750	0.0445	0.0117	0.3870
17	F:GCCCATGATCAGACTTCACA<br>R:TGAAGTGCTGGTGCCAGTTA	C/T	128	60.0	0.8528	0.4867	0.4286	-0.7523	0.0050	1.0000
18	F:TGTGGGTCAACATAGTGCGT<br>R:GTTCGCTATTTCCGGCTGTA	A/G	130	60.1	0.1613	0.1789	0.4881	0.0981	0.6387	0.4295
19	F:CATCTCGGTTTCCCCATCTA<br>R:TGGTGAGGATGGATACACCA	C/T	134	59.8	0.4756	0.3284	0.3191	-0.4483	0.2952	1.0000
20	F:TTTGAGCCATCAGATAGGGC<br>R:CATTGGTACTGTTTCCCGGT	C/T	135	59.9	0.0763	0.1627	0.5000	0.5310	0.6744	0.0127
21	F:ACCAATGCAGAAAAGCATCC<br>R:GGTCGCAAACCCACTATGTT	C/T	136	60.0	0.0385	0.2107	0.1167	0.8174	0.0406	0.0010
22	F:TATTGACCATTTGGTCGGGT<br>R:GGATGTCACGTGTGTCGTGT	A/G	137	60.3	0.5238	0.4615	0.3810	-0.1351	0.0216	0.6625
23	F:TGGCCAGTTTGCACTCATAC<br>R:AAGGCCGTGCAATAGGAATA	C/T	137	59.6	0.1250	0.2128	0.1222	0.4125	0.0744	0.0044
24	F:TACCACCGGAATCTCTTTGC<br>R:GAGCCCGTATTGGTAGTCCA	C/T	138	60.0	0.4494	0.4277	0.3108	-0.0508	0.0147	0.7731
25	F:CATGGTTTGACACACTTGGC<br>R:CGAATCACTTGTTGCAATGG	C/T	140	60.1	0.8421	0.4889	0.4608	-0.7224	0.1757	1.0000
26	F:CTTGAGTGCACACAGAACTGC<br>R:GGTTGATATTCCCGAGGGTT	A/G	140	59.8	0.2158	0.4912	0.4804	0.5606	0.0151	0.0000*
27	F:TCCCTTTAGCAACAAGGCAT<br>R:CAGAACCGAGACATTGAGCA	C/T	140	59.8	0.7880	0.4924	0.4487	-0.6003	0.0076	1.0000
28	F:CAACCATACAGGGACCTTGC<br>R:GCGCTATGGGTGCAGACTAC	C/T	141	60.6	0.6852	0.4763	0.4412	-0.4385	0.0364	0.9998
29	F:GTCCTCTGGGATGTCCTCAA<br>R:AATCACAGCTGTTGACGCAG	C/A	145	60.1	0.3846	0.3284	0.2308	-0.1712	0.0750	0.9334
30	F:AAGCGTGTCGTCTGGATCTT<br>R:CATGTCGTCTGCACCTTTGT	C/T	146	59.8	0.1117	0.4629	0.3667	0.7588	0.0027	0.0000*
31	F:ATTTCCCCCACTTCCAGTTC<br>R:AGCGCGGTATCATAATTTGG	C/T	150	60.1	0.0652	0.1403	0.4778	0.5351	0.7183	0.0051
32	F:ACCCAAACCACTGACATTGA<br>R:ACCTTCTGCTTCCCCTTGTT	A/G	150	59.5	0.3393	0.3045	0.1875	-0.1141	0.0005	0.7178
33	F:TTCAGGATACTCTCCACCGC<br>R:TTAACAGCTGAGTTGCCGAA	A/G	153	59.9	0.3452	0.3434	0.2213	-0.0051	0.0026	0.5883
34	F:CATTAGCATGTGCATACCGC<br>R:CGCACAAAGGAAGTTGTGAA	A/G	153	60.0	0.4350	0.3601	0.2340	-0.2079	0.0035	0.8486
35	F:TGGCATCCAAGATGTATTGC<br>R:GAATTCGACCACACGGAAAG	G/T	158	60.0	0.6333	0.4322	0.3163	-0.4653	0.0013	1.0000
36	F:ATCAACGATTCCAAACCCAG<br>R:AGCGAACCTTGTTCTGATGG	A/G	159	60.0	0.2250	0.4983	0.4796	0.5484	0.0017	0.0001*
37	F:CTGGAATTCCGAGTTCGTTG<br>R:CAAGCCAATCATCCATGTCA	A/G	159	60.6	0.1697	0.2567	0.4615	0.3388	0.4862	0.0625
38	F:CCTCTAAAAACTTGACGGATGG	C/T	160	60.0	0.1800	0.2845	0.1818	0.3673	0.0221	0.0098

	R:GGGTGACAGATCAGGGAGAA									
39	F:GGAAGGAAATGTGTTCCTGG<br>R:TTCAACGCTCTTGGGAGAAT	C/T	160	59.6	0.3328	0.4349	0.3265	0.2349	0.0168	0.0286
40	F:GAATTTTGAAATCAGCGGGA<br>R:GGTATGACCGCACCTTGTCT	C/T	161	60.0	0.3095	0.4518	0.4167	0.3149	0.0706	0.0063
41	F:TGCACGTAGATGGAAACCAC<br>R:TTTACAGGATCCGGACAACC	G/T	163	59.7	0.1786	0.4681	0.4762	0.6185	0.0626	0.0000*
42	F:ATTGCTGCTCATATTTCCGC<br>R:GCAGGACATTCAACAGATGC	A/G	165	59.7	0.4940	0.4332	0.3171	-0.1403	0.0025	0.7101
43	F:CAAGCGTCTCTGGTGACGTA<br>R:AAAGTGTGACGCACGTTGAC	G/T	165	59.9	0.4444	0.4877	0.4375	0.0888	0.0064	0.3298
44	F:TACAAGGGCGAGTTTAACGG<br>R:CTGTAATTGAGCAGCCGACA	C/T	169	60.1	0.3397	0.3885	0.3396	0.1255	0.1379	0.2856
45	F:AACAAGGGGAAGCAGAAGGT<br>R:TCCATACCTTCTTTGGACGG	C/T	169	60.0	0.4100	0.3919	0.4216	-0.0462	0.1942	0.6088
46	F:TGTTCGTGAAAAATTTGGGA<br>R:CAGGATTGCCAAGTTCATTG	A/G	169	59.1	0.2718	0.2337	0.1364	-0.1634	0.0052	1.0000
47	F:TGCATAAATCTGCAATGGCT<br>R:CTCCACTTAAACTGCTCGCC	G/T	170	59.7	0.5484	0.2960	0.2627	-0.8524	0.2562	1.0000
48	F:TGGCAGTCATCAGTGGTTGT<br>R:CTGTTTTGACGCTTGCACAT	C/T	172	60.0	0.4833	0.4693	0.3784	-0.0299	0.0004	0.5703
49	F:CAGGTTGTGTTGGTAGGTTTCA<br>R:CAGCCAGTGCAAGATCCTTA	A/G	173	59.5	0.5043	0.4540	0.3596	-0.1109	0.0132	0.6816
50	F:TACACGCTTTCCAGTCACCA<br>R:TTCCTCCTATGACAGCCCAC	C/T	174	60.2	0.2593	0.4691	0.3778	0.4474	0.0130	0.0203
51	F:ATTACCAAAAATGTCGCCCA<br>R:AATGCCACTTTAAAAGCCGA	G/T	174	60.0	0.2731	0.3919	0.4018	0.3032	0.1942	0.0128
52	F:TGCACGACTTTATGAACGGA<br>R:ATCGGTCCAACGATCAAAAC	A/G	175	60.0	0.3577	0.4582	0.3556	0.2194	0.0003	0.0402
53	F:TGCCACGTATCCCTCACATA<br>R:CACATCGACCAAATACGGAA	A/G	176	59.7	0.5307	0.3881	0.2692	-0.3676	0.0046	1.0000
54	F:CGGCGTAACGAGGTTTTCTA<br>R:GTTTTCGGGGATGACTCTCA	C/T	178	60.2	0.2036	0.2092	0.4634	0.0270	0.5811	0.2769
55	F:GACGGGAAGAATGTTCGGTA<br>R:TCACCACACAGCGCTAAATC	C/T	179	59.9	0.1667	0.4822	0.4333	0.6544	0.0181	0.0000*
56	F:TTGTGTCCTTGCATGTAGGC<br>R:GCTTAAAAGCGGAAGTGACG	C/A	180	59.9	0.2240	0.4867	0.4302	0.5398	0.0135	0.0000*
57	F:GGTTATCGACTGGGGGAGAT<br>R:ATGCATCTGATTCAACAGCG	C/T	183	60.0	0.9815	0.4997	0.4898	-0.9643	0.0003	1.0000
58	F:CCCAACCTCTGAAAACCGTA<br>R:TTGTGGATGACGTTCGGATA	C/T	184	59.9	0.4896	0.4324	0.3646	-0.1322	0.0725	0.5928
59	F:TCTCTGGGGTTTCCATCTTG<br>R:TGGATTTATTGTCCTGACGGA	C/T	184	60.2	0.3338	0.2778	0.1667	-0.2018	0.0012	1.0000
60	F:CGTTCTGTGCACCAACACTT<br>R:CCGCTTTCCTTCGACAATAA	C/T	184	60.0	0.4008	0.4071	0.4778	0.0154	0.1760	0.3973
61	F:TCCTGATTATTTTCCGCTGC<br>R:GACGAATTCTTTTCGAACGC	C/T	185	60.0	0.4188	0.4775	0.4528	0.1230	0.0329	0.1928
62	F:CCGTCCAAAGAAGGTATGGA<br>R:TGCGGATCACTTGTCATAGC	A/G	185	59.9	0.2321	0.2050	0.1161	-0.1322	0.0008	1.0000
63	F:CCTAGTTCTCGTCTGGCTGC	C/T	185	60.1	0.4881	0.4630	0.3723	-0.0543	0.0053	0.6435

	R:TTCTGGACCGGCTATTTCAC									
64	F:CCAGGCGTTGTGCAAATACT<br>R:CTCGACAGTCTGCAAGATGC	C/T	186	60.4	0.3278	0.3813	0.4512	0.1404	0.2344	0.0717
65	F:TGCATTCTTTATGTGACTGCG<br>R:TGTTTCCCCTTTCAGCTGTC	A/G	188	60.1	0.5417	0.4306	0.3300	-0.2581	0.0312	0.9821
66	F:GATCTGCGTTTTACATAAACCAA<br>R:GTTGCAAATGCAGGTTTTGA	A/G	189	59.0	0.3412	0.3716	0.2456	0.0817	0.0025	0.4198
67	F:TTATGGCGCAGTAAGACACG<br>R:TAAGAATTCCCTGCCCCTCT	C/A	191	60.0	0.2990	0.2511	0.1475	-0.1909	0.0127	1.0000
68	F:GGCACGTTCAGGAAGACATT<br>R:TCTCAACGGTTTGTCGTCAC	A/G	192	59.9	0.1123	0.1827	0.1053	0.3853	0.0946	0.0495
69	F:ACATGTCAATCGGCAATGG<br>R:ACGCAACTGCTTTTGCATC	C/T	196	60.2	0.6238	0.4456	0.4028	-0.4001	0.0576	0.9971
70	F:CGACCTTGTCTATTTCGGGA<br>R:CTGCTCATAGCATTATGCCG	C/T	196	59.8	0.2156	0.2653	0.2000	0.1872	0.1619	0.2046
71	F:AGCAACGTACACTGCGATTG<br>R:GCAACCTTAACCAGTCAGCC	A/G	197	59.8	0.0690	0.1775	0.0984	0.6115	0.0158	0.0000*
72	F:CAAGACGCCAAGGTGAAGTC<br>R:TGACATGGATCAACTGCACA	C/T	199	60.2	0.4165	0.3653	0.2407	-0.1401	0.0000	0.8654
73	F:CACACGGGTTCTACCTTGCT<br>R:GCCGGATCTACAGGAAACAA	C/A	200	60.1	0.2366	0.2318	0.4667	-0.0206	0.5362	0.4297
74	F:TTACATCATGTTCCCCACCA<br>R:CACACTCTTCAGCACGCTATG	G/T	200	59.6	0.0200	0.2751	0.1667	0.9273	0.0016	0.0000*
75	F:TTCAAACATGCATTGTCGGT<br>R:TTGGGTCCGGATGTAATGAT	A/G	201	60.0	0.1852	0.4348	0.3269	0.5741	0.0093	0.0000*
76	F:GCTGTGGGCATTGTTGACTA<br>R:TTCATGGCCAAACATCAACT	C/T	202	59.3	0.4467	0.4681	0.3721	0.0457	0.0009	0.5572
77	F:ATATGTCGAATCCCAGCCAC<br>R:AAGCTTTGTGTACACGTGCG	A/G	203	59.9	0.1071	0.4822	0.4068	0.7778	0.0018	0.0000*
78	F:AGGAATTATTGAACCGGCAA<br>R:GGCATCTGCAAAGTCAACTG	C/T	204	59.4	0.2174	0.2757	0.1667	0.2116	0.0169	0.0053
79	F:CCGATGAAAGGGATGACAGT<br>R:ATTTGGAACACGCAAATGGT	C/A	204	60.1	0.1522	0.3439	0.2179	0.5575	0.0360	0.0271
80	F:GTGTATGACAGTTCGCCACG<br>R:GCGTCATAAACCGTCTCCAT	A/G	204	60.1	0.6038	0.4872	0.4804	-0.2395	0.0244	0.9724
81	F:ATGCCACGAAGCATTTTGTT<br>R:CCGGATGTTTTGTGTTGTTG	C/T	206	60.2	0.0702	0.0677	0.0351	-0.0364	0.0000	1.0000
82	F:GGCATCAAATTTCACGTCGTA<br>R:GAATTCCAGCCAATTTGTTCA	A/G	206	60.4	0.3564	0.4963	0.5000	0.2820	0.0073	0.0172
83	F:TCATCAAAAAGGGCAAGGTC<br>R:CCATTCTGAACACTTTCCCG	C/A	209	60.3	0.0000	0.2479	0.2273	1.0000	0.2941	0.0000*
84	F:GTTTGCAATCCCTTGCATCT<br>R:TTTTCGTTTTGATTCTTCCCA	G/T	209	59.8	0.0185	0.3222	0.2021	0.9425	0.0000	0.0000*
85	F:TTTATTGCGAGCCTAGCGTT<br>R:GTATACGCGAACGGGTGAAT	A/G	209	59.9	0.3705	0.3665	0.4554	-0.0110	0.2650	0.6582
86	F:CCTTATCAGCCTTGCTTTGG<br>R:GATCCCGTCCTCTTTCTTCC	G/T	210	59.9	0.1875	0.4249	0.3438	0.5587	0.0582	0.0002*
87	F:GTTGGGCAGATTTCACTGGT<br>R:TTGAAAACGAGGAGGCACTT	G/T	210	59.9	0.1503	0.1860	0.4661	0.1916	0.6237	0.1916
88	F:ACAGTGGGGAACACTTTTCG	C/T	210	60.0	0.3060	0.4306	0.4000	0.2894	0.0934	0.0106

R:CAACATCACGACAATCAGGG

PHWE, the P-values for Hardy–Weinberg equilibrium test (*$p < 0.05/88 = 0.0006$)

Text S1. GATK Filter description

The Unified Genotyper module in GATK (version 3.4-46) was used for SNP detection (parameter -t Unified Genotyper-glm BOTH -nt 8) -filter "QD < 4.0" || "FS > 50.0" || "MQ < 40.0", -G_filter "GQ < 20 -filter" Multiallelic (Non allelic genotype). The detected variants were annotated using ANNOVAR (version 2). QD: Variant call confidence normalized by depth of sample reads supporting a variant. FS: (Fisher Strand): Fisher's exact test assesses the likelihood that the current variant is a strand bias. GQ: Genotype Quality. MQ: (RMS Mapping Quality) All comparisons read the root mean square of the comparison mass to determine the average comparison mass of a loci.
